# Overcoming Analytical Challenges for the Detection of 27 Cyanopeptides Using a UHPLC-QqQ-MS Method in Fish Tissues

**DOI:** 10.3390/toxins17120580

**Published:** 2025-12-02

**Authors:** Audrey Roy-Lachapelle, François-Xavier Teysseire, Christian Gagnon

**Affiliations:** Environment and Climate Change Canada, Montréal, QC H2Y 2E7, Canada

**Keywords:** cyanopeptides, cyanotoxins, cyanobacteria, harmful cyanobacterial blooms, fish tissue analysis, UHPLC-QqQ-MS, microcystins, anabaenopeptins, microginins, aeruginosins

## Abstract

The increasing occurrence of harmful cyanobacterial blooms in freshwater ecosystems poses important risks to aquatic organisms and human health due to the production of bioactive secondary metabolites such as cyanopeptides. While analytical methods for microcystins are well developed, there is a notable lack of validated protocols for the broader spectrum of cyanopeptides in biota. This study presents the development and validation of a robust UHPLC-QqQ-MS method for the simultaneous extraction, cleanup, and quantification of 27 cyanopeptides, including microcystins, anabaenopeptins, microginins, aeruginosins, aeruginoguanidine, and nodularin, in fish muscle, liver, and whole fish tissues. Comprehensive optimization was conducted to minimize matrix effects and analyte losses during every step of sample preparation. The method demonstrated generally high recoveries (28–98%), good precision (RSD < 20%), and sensitivity, with MQLs below 0.5 ng g^−1^ for most analytes. Microginins posed analytical challenges due to their amphiphilic structure, which contributed to significant losses during filtration and extraction; the reasoning is discussed. Application to wild fish collected after a mass mortality event revealed no detectable cyanopeptide contamination but confirmed the method’s suitability for comprehensive detection. This represents an important advancement in cyanopeptide analysis, offering a valuable tool for environmental risk assessment and food safety evaluation related to harmful cyanobacteria.

## 1. Introduction

The proliferation of cyanobacteria in freshwater ecosystems has become a major environmental concern due to its disruption of aquatic ecosystem functions and services [1]. These microorganisms can dominate water bodies by outcompeting other species and disrupting food webs [2,3]. Additionally, their production of harmful secondary metabolites poses significant risks to aquatic ecosystems, wildlife, and human health [4]. These bioactive compounds are highly diverse, exhibiting a wide range of physico-chemical characteristics and structural variations that result in diverse toxicological effects. They include well-known cyanotoxins such as microcystins, nodularins, anatoxins, saxitoxins, and cylindrospermopsins, alongside less-studied bioactive compounds such as anabaenopeptins, aeruginosins, cyanopeptolins, microginins, and cyclamides [5,6]. These families, called cyanopeptides, are mainly produced by genera such as *Microcystis*, *Dolichospermum*, *Planktothrix*, *Nodularia*, and *Oscillatoria* and differ structurally. They include cyclic and linear peptides with variable amino acid residues, methylations, and halogenations, which contribute to their chemical complexity and diverse ecological roles [5]. These cyanopeptides can enter and circulate through the aquatic food webs, impacting organisms at different trophic levels [7]. Fish, a key actor within these food webs, can bioaccumulate these compounds, making their analysis crucial for understanding the ecological impacts of harmful cyanobacterial blooms and assessing human health risks [8,9].

Cyanopeptides represent structurally diverse families of compounds, including hundreds of congeners identified to date, and new structures are still being discovered [5,6]. Chemical structures of cyanopeptides of interest in this study are presented in Figure S1 in Supplementary Materials. Microcystins, the most extensively studied family of cyanopeptides, include over 300 characterized congeners identified to date [6]. They are inhibitors of serine/threonine protein phosphatases, disrupting cellular processes and causing hepatotoxicity [10]. Chronic exposure to microcystins has been linked to liver damage, tumor promotion, and potentially carcinogenic effects in humans [11]. Nodularins, which share structural similarities with microcystins, exhibit comparable inhibitory effects and similar hepatotoxicity [12]. Anabaenopeptins, aeruginosins, cyanopeptolins, and microginins also display relevant bioactivity (e.g., inhibition of protease or phosphatase), but their ecological and toxicological significance remains insufficiently characterized, further underscoring the need to include these compounds in ecotoxicological assessment [5].

Despite the diversity of these cyanobacterial secondary metabolites, most analytical developments have mainly focused on microcystins, resulting in a notable knowledge gap regarding the occurrence, toxicity, and environmental dynamics of other cyanopeptides [1]. Detecting these cyanopeptides with higher abilities is therefore essential to improve risk assessment and ecosystem monitoring, as they may contribute to overall toxicity and ecological effects that remain poorly characterized but increasingly observed in cyanobacterial blooms [5]. More specifically, the lack of multi-cyanopeptide analytical methods using mass spectrometry for fish matrices represents a significant limitation in the risk assessment of toxic cyanobacterial blooms. The limited availability and accessibility of certified standards for most of these compounds are a major challenge for the inclusion of a broader range of compounds [1,6]. In this regard, most methods have been developed for the analysis of bioaccumulated microcystins, focusing on separate extractions of liver, muscle, or other organs in fish as well as mice [13,14,15,16,17,18,19,20,21,22,23,24]. Additionally, few studies have incorporated multiple families of cyanotoxins in biota analysis. Notably, Turner et al. and Diez-Quijada et al. included nodularin and cylindrospermopsin in their studies; however, their methods were targeting only mussels and algae [25,26]. Another study has explored multi-cyanotoxin extraction in fish, but the range of cyanopeptide classes considered and the level of detail provided on extraction performance across structurally diverse families remain limited [21].

Analyzing these compounds in fish is challenging due to their low concentrations and the complexity of the tissue matrix with high and varying lipid and protein contents, which can cause variable matrix effects due to ion suppression in mass spectrometric analyses [8,27]. For example, microcystin congeners cover a broad polarity range (e.g., MC-LR is moderately polar, MC-RR is highly polar, and MC-LF/LY is more hydrophobic), which directly influences extraction performance and recovery in both muscle and liver. Efficient extraction is also impacted by a wide polarity range of cyanopeptides, complicating method optimization. The lack of validated analytical approaches for the broader spectrum of cyanopeptides limits the understanding of how parameter changes affect the results [28]. Detecting microcystins in complex matrices such as liver poses unique analytical challenges because they can form conjugates with cellular thiols, particularly cysteine and glutathione, in liver tissue [29]. These conjugates complicate extraction and detection, and one solution is the Lemieux oxidation, a reaction that releases cysteine-bound microcystins from liver tissue [30]. The oxidation targets the Adda (3-amino-9-methoxy-2,6,8-trimethyl-10-phenyl-deca-4,6-dienoic acid) moiety of microcystins, releasing the MMPB (2-methyl-3-methoxy-4-phenylbutyric acid) fragment, which is common to all congeners and allows the analysis of total microcystins [30,31,32]. While effective for total microcystin measurement, this method is not specific for individual congeners and is unsuitable for detecting other cyanopeptides.

This study addressed a critical need for methods enabling the detection of structurally diverse cyanopeptides to improve ecological monitoring, research, and risk assessment. An analytical method was developed for detecting a broad range of cyanopeptides, including microcystins, anabaenopeptins, microginins, aeruginosins, aeruginoguanidine, and nodularin, in fish muscle and liver. By including compounds with a wide range of physico-chemical properties, this study enabled the investigation of key parameters influencing extraction and analysis. A comprehensive analysis of fish muscle, liver, and whole fish provided a robust approach for the evaluation of cyanopeptide bioavailability. This is critical for understanding their transfer through the food web, offering better insights into ecological and human health risks. Additionally, identifying compound-specific losses, particularly for the amphiphilic microginin, helps define analytical bottlenecks and supports future method development. Therefore, several methodological choices used in previous cyanopeptide studies, such as sample containers, non-optimized sonication times, number of extraction cycles, or solvent mixtures, were critically evaluated in this work, as these steps are known to cause substantial and often unreported analyte losses. The optimized method was finally applied to wild fish collected after a mass mortality during a cyanobacterial bloom to investigate the presence of cyanopeptides and the method’s suitability for comprehensive detection. By expanding the scope of detectable compounds and refining extraction protocols, this work contributes to more accurate cyanopeptide exposure assessments, informing on their ecological impacts as well as implications for food safety.

## 2. Results and Discussion

### 2.1. Extraction Development in Muscle

Since various extraction conditions have been reported in the literature for fish muscle and liver (primarily for microcystin (MC) quantification and limited for other cyanopeptides) with little consensus or a limited number of compounds studied, optimization of extraction parameters was studied. Ultrasonic-assisted extraction was initially performed on fish muscle tissue to take account of the free form of cyanopeptides. This approach allowed us to evaluate each extraction parameter and identify potential losses associated with complex matrix interactions and obtain robust and reproducible quantification results.

#### 2.1.1. Impact of Polypropylene Adsorption

The well-documented adsorption of microcystins onto polypropylene (PP) can strongly affect analyte recovery [33,34]. Because cyanopeptides can exhibit similar hydrophobic interactions, understanding the PP-related losses is important where considerable adsorption can lead to an underestimation or even no detection of these compounds. To evaluate these effects, 10 µg L^−1^ of analytes were spiked into distilled water, vortexed for 30 s, sonicated for 10 min, and submitted to solid-phase extraction (SPE). Figure S2 presents losses in percentages, with values ranging from −27% to −98%, when samples were spiked in a PP Falcon^®^ tube (Fisher Scientific, Whitby, ON, Canada). The most apolar microcystins (LW, LY, LF, and LA) showed higher losses exceeding 90%, and all congeners showed 66% losses on average, which is in accordance with a previous study [33]. Microginins (MG) and aeruginoguanidine (AGU) experienced > 50% losses, while anabaenopeptins (AP) were less affected (31% on average). Comparable losses were obtained in spiked fish muscle (Figure S3), confirming that PP-related adsorption can compromise interpretation of environmental concentrations. To minimize this bias, glass tubes were thus selected to reduce hydrophobic adsorption for all extraction steps [33,34].

#### 2.1.2. Sonication Time

**Figure 1:**
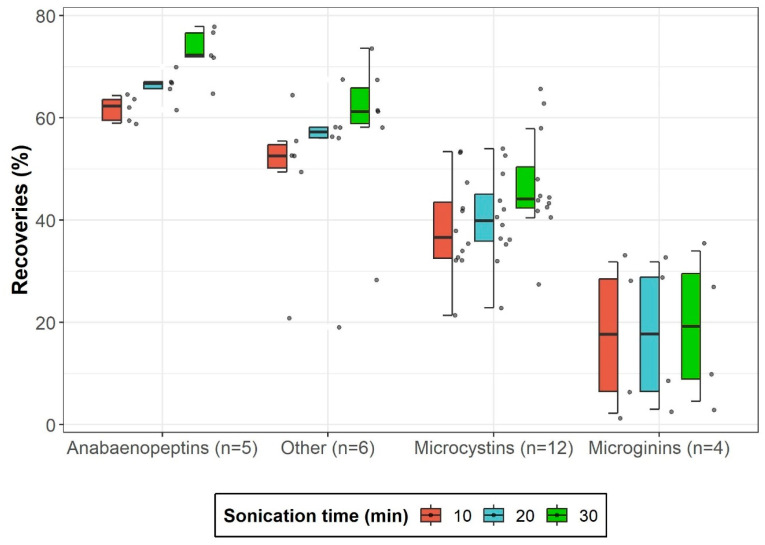
Boxplots illustrating ultrasonic-assisted extraction recoveries for four cyanopeptide groups across three sonication times. The value *n* represents the number of analytes included in each group. The median is denoted by a horizontal line within each box. Data points beyond the boxplots represent statistical outliers, while whisker extensions correspond to 1.5 times the interquartile range.

Sonication time was investigated to optimize extraction efficiency while minimizing its duration using fish muscle spiked at 10 ng g^−1^. As shown in Figure 1, recoveries generally increased with longer sonication for 26 of 27 cyanopeptides. Statistical analysis using one-way ANOVA (analysis of variance) revealed significant differences (*p* < 0.05) for six compounds (MG-690-ME, MG-FR1, MG-FR2, MC-LR, ferintoic acid A (FA-A), and AP-J) and trends toward significance for twelve others (*p* < 0.1). The large standard deviations likely contributed to the lack of statistical significance for these twelve compounds (0.1 > *p* > 0.05). Post-hoc Tukey tests indicated a significant improvement between 10 and 30 min for all compounds, but only for two between 20 and 30. No significant difference was found between 10 and 20 min. Increasing sonication time from 10 to 30 min resulted in an average recovery increase of 8%, with a maximum increase of 19% for AP-J, and particularly improved recovery for microcystins and anabaenopeptins. The gain on microgins was minimal with 2%. Based on these results, 30 min proved to be an acceptable time for sonication, ensuring better recoveries.

#### 2.1.3. Extraction Cycle Number

**Figure 2:**
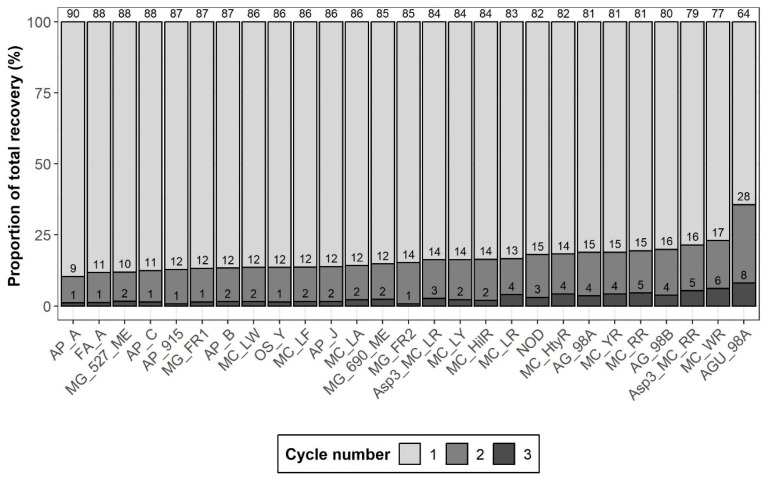
Proportional contribution of individual cycles to the total recovery. The percentage recovery for each cycle is annotated above its respective bar.

The number of extraction cycles also affects extraction efficiency, with three cycles theoretically maximizing extraction efficiency. However, additional cycles increase processing time and co-extract more matrix components, potentially impacting the analyte detection. Therefore, one to three cycles were tested to determine the optimal conditions using fish muscles spiked at 10 ng g^−1^. As presented in Figure 2, recoveries improved for 26 of 27 analytes when a second extraction was added, with a 10% increase for most compounds and reaching 28% for AGU-98A. A third cycle led to minimal improvements (<3% for 16 out of 27) while AGU-98A showed an additional 8% increase. Overall, the third extraction cycle had minimal impact on analyte recovery but increased the amount of extracted matrix, as observed visually. Therefore, two extraction cycles were performed for optimal recovery and method efficiency.

#### 2.1.4. Liquid-Liquid Extraction Conditions Evaluation

**Figure 3:**
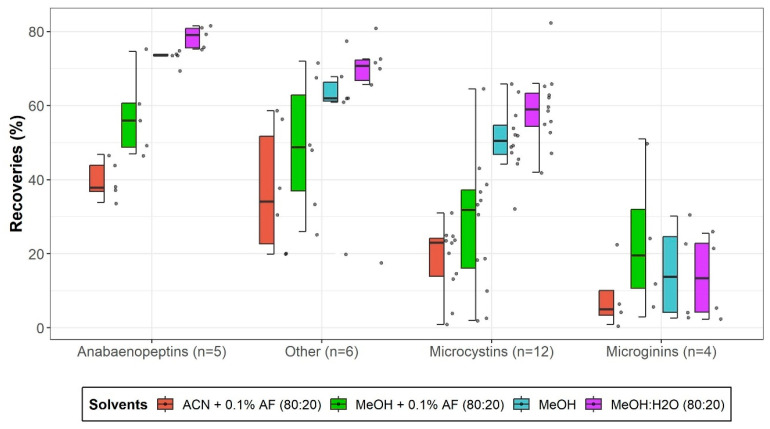
Ultrasonic-assisted extraction recoveries for four cyanopeptides groups across four extraction solvent conditions. The value n represents the number of analytes included in each group. The median is denoted by a horizontal line within each box. Data points beyond the boxplots represent statistical outliers, while whisker extensions correspond to 1.5 times the interquartile range.

Various solvent mixtures were evaluated to determine the optimal conditions for cyanopeptide extraction from fish muscle based on reported approaches [15,19,35]. Figure 3 illustrates the impact of solvent composition on the recovery across cyanopeptide classes. Polar microcystins such as MC-RR and [Asp3]MC-RR exhibited improved recoveries (78% and 82%, respectively) when using methanol:water (MeOH:H_2_O—80:20 *v*/*v*), likely due to enhanced solubility in aqueous conditions. Conversely, hydrophobic microcystins such as MC-LF, MC-LW, and MC-LY showed reduced recoveries, with losses ranging from 25% to 44% under acidic conditions (MeOH:H_2_O + 0.1% formic acid). This reflects pH-dependent changes in charge state that reduce solubility and potentially promote degradation during sonication [22,36]. Anabaenopeptins and aeruginoguanidine displayed moderate recovery variability (60% and 75%, respectively), with MeOH:H_2_O generally outperforming pure MeOH. Microginins, including MG-527 and MG-690, demonstrated notable improvements in recoveries (85% and 88% under acidic conditions), likely due to reduced logD values, which enhance solubility.

The use of acetonitrile (ACN) + 0.1% formic acid (80:20 *v*/*v*) had minimal impact on most analytes (<2% gain), though hydrophobic microcystins were slightly benefited (up to +12.8%). Overall, MeOH:H2O (80:20 *v*/*v*) was selected as the optimal solvent mixture, providing an average recovery of 79% across all analytes and offering a balanced extraction for compounds differing in polarities while minimizing losses.

To assess the influence of solvent polarity on analyte recovery, a hexane liquid–liquid purification step was tested as commonly used to remove lipophilic interferences before further extraction steps [15]. As presented in Figure S4, hexane caused significant losses for 17 of 27 analytes (up to 47%), with microcystins and microginins being the most affected, with average losses of 26% and 29%. Only MC-WR and MC-LR were minimally impacted, showing losses of 15% and 6%, respectively. Losses could be attributed to reduced solubility and/or potential sorption to surfaces for polar microcystins, while microginin losses can reflect partial partitioning into hexane due to their aliphatic chains, although this was not specifically evaluated. Anabaenopeptins and aeruginoguanidine experienced moderate losses of 14–15%. Given these findings, the hexane purification step was removed, and instead, samples were centrifuged at −9 °C to precipitate proteins and lipids via freezing in accordance with a previous study [21].

#### 2.1.5. Methanol Percentage in Sample

Ultrasonic-assisted extraction was performed using 80% MeOH, and this proportion must be reduced prior to the SPE loading to ensure effective retention. Since an additional drying step is time-consuming and may lead to further losses, dilution of the supernatant was evaluated as a simpler alternative. As illustrated in Figure S5, ten microcystins and FA-A were highly affected by methanol content, with decreased recoveries when 5% MeOH was added to 100 mL extract. Their higher polarity reduces their retention on SPE sorbent in the presence of MeOH, explaining the observed losses. In contrast, less polar cyanopeptides were less affected and were more efficiently retained. Therefore, to avoid compromising microcystin recoveries, an evaporation step was added to reduce MeOH to <2% before the SPE step, ensuring consistent loading across cyanopeptide classes.

#### 2.1.6. Filtration Step Prior to Analysis

Prior to analysis, a prefiltration step was incorporated to mitigate the effects of potential matrix suspensions on the analytical performance. Literature supports the use of GHP (hydrophilic polypropylene) filters due to their broad chemical compatibility and reduced sorption characteristics [37,38]. As presented in Figure S6, 21 of 27 analytes showed minimal losses (<10%), demonstrating its suitability for most compounds. However, six analytes, primarily microginins and aeruginosins, exhibited higher losses up to 50%, but average recoveries were 77% and 81%, respectively. These losses were likely attributable to interactions between their amphiphilic structures and the filter material causing sorption. Despite these losses, the prefiltration was retained as an essential step for removing particulate matter, and its optimization should be considered to balance recovery with matrix cleanliness.

### 2.2. Extraction Development in Liver

Using the optimized extraction conditions developed for fish muscle tissue as a foundation, additional steps were tested to adapt and optimize the protocol for fish liver. Given the challenges posed by the liver matrix, for instance the covalent binding of microcystins to cysteine residues, it was essential to evaluate the efficiency and recovery of cyanopeptide extractions under these conditions [18,29,39].

#### 2.2.1. Optimization of Equilibration Time

**Figure 4:**
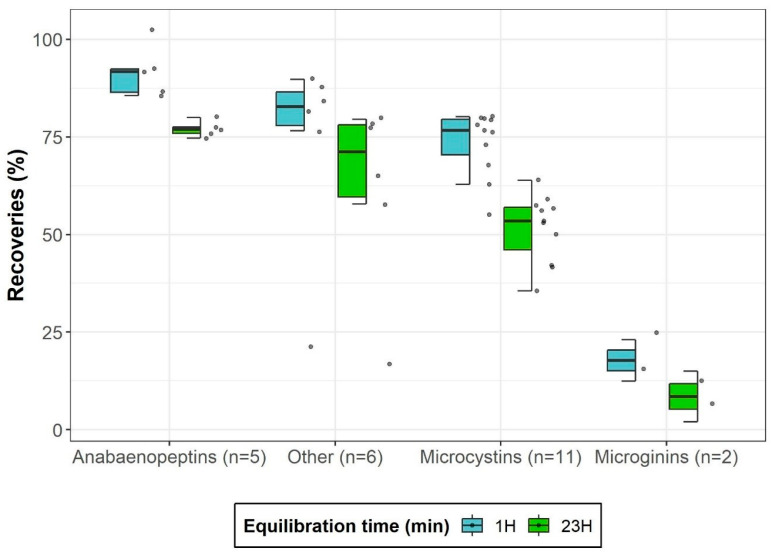
Ultrasonic-assisted extraction recoveries in liver for four cyanopeptide groups after two equilibration times. The value n represents the number of analytes included in each group. The median is denoted by a horizontal line within each box. Data points beyond the boxplots represent statistical outliers, while whisker extensions correspond to 1.5 times the interquartile range.

Equilibration time influences compound release from liver tissue due to potential compound sorption and covalent binding. Microcystins can form irreversible covalent binding between the Mdha (*N*-methyldehydroalanine) residue and a cysteine thiol (-SH) group in the active site of PP1/PP2A [39]. Previous studies reported up to 60% recovery losses for MC-LR, MC-RR, and MC-LA after 20 h of equilibration [18,40]. Our results indicated greater variability in recoveries after 1 h of equilibration (Figure 4), suggesting ongoing covalent-bond formation. After 23 h, recoveries stabilized for most analytes except for MC-RR and [Asp3]MC-RR. Median losses for microcystins reached 29%, with substantial variability among congeners, indicating that the tendency for covalent bonding is structure dependent. Except for microginins and FA-A, all other analyte classes showed similar mean recoveries and standard deviations between 1-h and 23-h equilibration times, suggesting that a covalent bond formation with liver matrix components is unlikely for these compounds. Although the Lemieux oxidation can quantify total microcystins via MMPB and effectively extract this compound from liver tissues, it does not preserve congener-specific information and exclude other cyanopeptide families [41]. This highlights the need for improved direct-extraction approaches for risk assessment, as this developed method enables the detection of a broader range of cyanopeptide classes.

#### 2.2.2. Chromatographic Gradient Modifications

Using the chromatographic conditions optimized for fish muscle, the presence of peak splitting of MC-RR was observed in liver extract (Figure S7), likely due to co-eluting proteins [27]. To address this, the gradient was modified by extending the elution window by 4 min (Figure S7A), aiming to separate matrix components from target analytes. Important signal loss was observed for analytes eluting between 5.10 and 5.20 min, leading to a second adjustment of elution time from 0.10 min to 0.15 min. Although signal suppression in muscle was observed, peak splitting was absent in that matrix. To ensure complete matrix elution, the ACN 100% plateau was extended by 1 min, improving overall separation. The first chromatographic conditions for muscle and adapted conditions for liver are presented in Figure S7A. As shown in Figure S8, the new chromatographic gradient also reduced matrix suppression for most analytes. This confirms the improved separation and reduced interference under the adjusted conditions.

### 2.3. Validation

#### 2.3.1. Recovery and Matrix Effects

Recoveries varied between muscle and liver samples, with muscle yielding generally higher values. Liver samples exhibited stronger matrix suppression due to the higher lipid content, enhancing analyte partitioning and/or interfering with ionization [42]. Three microginins (MG-690-ME, MG-FR1, and MG-FR2) were undetected in both tissues due to extreme matrix suppression (−91% to −58%), likely reflecting interactions between their aliphatic chains and lipid droplets, which were precipitated during freezing and centrifugation. Recoveries ranged from 28% to 98%, with slightly better performance at high QC (median 74% vs. 80%, mean 73% vs. 77%). MC-RR (−52%), AGU-98A (−29%), and MC-LR (−20%) showed concentration-dependent losses reflecting low-level adsorption, matrix interference, or protein adduct formation [29,34,42].

Matrix effects strongly influenced results: 13 analytes at high QC (quality control) and 14 at low QC concentrations exhibiting signal suppression. More specifically, AGU-98A, MG-690-ME, MC-RR, and [Asp^3^]MC-RR (>80% suppression), all eluting at 5.20–5.26 min, co-eluted with matrix components, reducing ionization efficiency. Microginins displayed signal loss > 20%, consistent with their amphiphilic structure [43]. Conversely, eight analytes exhibited signal enhancement, with MC-HilR, MC-WR (+28% at high QC), and AP-B (+32% at low QC), which suggests possible ionization facilitation from matrix components. Despite these variations, matrix effects between QC levels were statistically similar (*p* = 0.99), confirming the reproducibility.

#### 2.3.2. Precision, Accuracy, and Sensitivity

**Figure 5:**
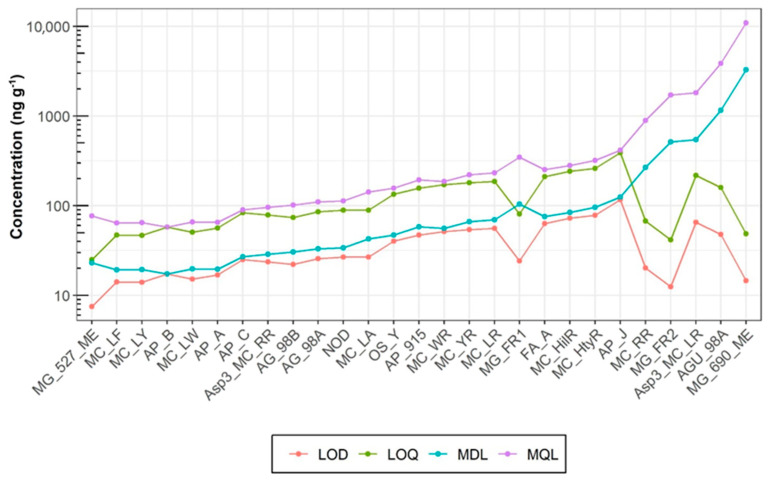
Comparative analysis of detection (LOD), quantification (LOQ), method detection (MDL), and method quantification (MQL) limits obtained for the developed extraction method applied to 27 cyanotoxins.

Matrix-matched correction with pooled samples was applied to account for recovery differences and matrix effects, ensuring accurate quantification of analytes across different tissue types. Most analytes displayed relative standard deviations (RSD) below 10%, indicating good repeatability, with higher variability for [Asp3]MC-RR (RSD 12.09% in muscle, 18.43% in liver) and MG-527-ME (RSD 19.85% in muscle), likely due to strong matrix effects and low recoveries. Accuracy showed generally acceptable limits (better than 20%), though MC-RR in liver showed a high positive bias (24.52%), while MG-690-ME in muscle had a strong negative bias (−37.45%). Matrix-matched correction helped mitigate signal variations, improving overall method reliability. Despite these challenges, the method demonstrated robustness for quantifying cyanopeptides in fish tissue, particularly in muscle, where variability was lower compared to liver. Figure S9 shows the calibration curves compared in the solvent, the matrix, and the matrix-matched corrected curve for key compounds in the study where the results of signal suppression and enhancement can be observed. All linearity values were satisfactory, with the coefficient of determination (R^2^) exceeding 0.998 (Table S1). Method limits of quantification (MQLs) were below 0.1 ng g^−1^ for 8 analytes and below 0.5 ng g^−1^ for 19 analytes (Figure 5), where the compounds showing the highest quantification limits (LOQ > 1 ng g^−1^) were the most affected by matrix effects (MG-690-ME, AGU-98A, and [Asp3]MC-RR). Analytes with a large gap between LOQ and MQL tend to have low absolute recoveries, which may be due to matrix effects or incomplete recovery.

#### 2.3.3. Microginins

Microginins posed analytical challenges due to their amphiphilic structure, resulting in important extraction losses and strong matrix suppression, particularly in liver. Filtration also increased losses due to sorption onto filter materials, a negligible phenomenon for other analytes. To enhance recovery, optimizing pH into acidic conditions can improve solubility and stability; while modifying solvent polarity and incorporating alternative filtration materials can minimize compound loss. Given their unique behavior, microginins may require dedicated analytical workflows for accurate quantification in biota.

### 2.4. Application in Environmental Samples

The analysis of wild fish samples in this study aimed to assess the presence of various cyanopeptides. The method was applied to real environmental samples, specifically whole fish, providing a realistic evaluation of its performance under natural conditions. The results showed that the cyanopeptides analyzed were below MDLs, indicating little to no detectable contamination in the samples. However, these results must be interpreted in relation to the MDLs, as the actual concentrations may be lower than the detectable range. Potential degradation pathways (e.g., microbial degradation, transformation during post-mortem handling) can also contribute to non-detections and should be considered when evaluating field results. Therefore, with no confirmed detection, the study was unable to conclusively link toxic cyanobacteria exposure to the observed rainbow smelts’ mortality.

To validate the method in whole fish, yellow perch samples were spiked at two concentrations (QC_low_ and QC_high_—see Section 4.5) and analyzed according to the developed method. Results demonstrated high recovery (80–120%), with matrix effects ranging from 50% to 60% depending on tissue type. The method showed excellent linearity (R^2^ values from 0.998 to 0.999) and good reproducibility, with coefficients of variation below 20%. Accuracy was within acceptable limits, with deviations from −7.9% to +6.8% for low concentrations and −8.0% to +1.9% for high concentrations. The method proved effective for detecting and quantifying cyanopeptides and performed better with whole fish samples compared to liver tissues, showing more consistent recoveries and lower matrix effects. Whole fish provided a more reliable and straightforward analysis, especially for small fish, making the method easier to apply and more efficient for cyanopeptide detection. These analytical performances highlight the method’s relevance for ecotoxicology and food safety, where including a wider range of cyanopeptides is essential to evaluate exposure in aquatic organisms and potential transfer through the food web, considering their high prevalence in cyanobacterial blooms [5].

## 3. Conclusions

This study addressed a critical gap in cyanopeptide analysis by developing and validating a comprehensive and reproducible method for the simultaneous quantification of 27 cyanopeptides in fish muscle, liver, and whole fish tissues. The method overcomes key analytical challenges, including analyte losses due to sorption, covalent binding, and matrix effects, through systematic optimization of extraction and cleanup parameters. Recoveries were generally high and consistent across most analyte classes, with matrix-matched corrections effectively accounting for ion suppression and enhancement. The method’s robustness was demonstrated through its successful application to real fish samples, confirming its reliability under environmental conditions. While liver matrices presented stronger challenges due to higher lipid content and potential for protein-adduct formation of microcystins, whole fish analysis emerged as a practical and efficient approach for cyanopeptide monitoring. It should be underlined that microginins exhibited the lowest recoveries, indicating the need for further targeted method development. This investigation showed that re-evaluating each extraction step helped resolve several limitations reported in earlier protocols, resulting in more consistent recoveries across the different cyanopeptide classes compared with previous methods. Overall, the method offers a powerful tool for the integrated analysis of diverse cyanopeptides, supporting ecological studies, food safety evaluations, and the monitoring of freshwater ecosystems impacted by toxic cyanobacterial blooms.

## 4. Materials and Methods

### 4.1. Chemicals and Standard Solutions

All analyte standards had a purity grade > 90%: Microcystins (MC-LR, [Asp3]-LR, [Asp3]-RR, -RR, -YR, -LA, -WR, -LW, -LF, -LY, -HtyR, and -HilR) and Anabaenopeptins (AP-A, -B), Microginin 527 methyl ester (MG-527-ME), MG-690-ME, and Nodularin-R (NOD-R) (purity ≥ 95%) were purchased from Enzo Life Science (Farmingdale, NY, USA). AP-C, AP-J, AP-915, Ferintoic Acid A (FA-A), Oscillamide Y (OC-Y), MG-FR1, MG-FR2, Aeruginosins (AG-98A, -98B), and Aeruginoguanidine 98A (AGU-98A) (purity ≥ 90%) as bioreagents were purchased from Cyano Biotech GmbH (Berlin, Germany). Methanol (MeOH), water (H_2_O), hexane, acetonitrile (ACN), and formic acid (FA) were LC-MS grade and purchased from Fisher Scientific (Whitby, ON, Canada). Nitrogen used for drying was acquired from Messer (Montreal, QC, Canada) and was of grade 5.0.

Individual stock solutions were prepared by dissolving 100 µg of solid standards in 1 mL of 80% MeOH in water and stored at −20 °C for up to twelve months. The integrity of these stock solutions was assessed monthly by injecting diluted solutions of each standard to monitor any degradation or methylation. The intensity of standards in the diluted solution was tracked, ensuring variability remained below 20%. Primary working solutions were prepared at a concentration of 1 µg L^−1^ for targeted cyanotoxins by diluting aliquots of individual stock solutions with water. Secondary working solutions were prepared daily by further dilution with water to achieve concentrations between 1 and 250 µg L^−1^, with a final MeOH content of 10%. Primary and secondary working solutions were stored at −20 °C for up to three months.

PALL wwPTFE acrodisc^®^ syringe filters of 25 mm and with a pore size of 0.22 µm were purchased from Waters (Franklin, MA, USA). Oasis HLB cartridges (30 µm, 200 mg, 6 mL) for solid-phase extraction (SPE) were obtained from Waters (Franklin, MA, USA). Amber glass tubes of 40 mL and 10 mL centrifuge tubes were purchased from Thermo Fisher ScientificTM (Mississauga, ON, Canada). Polyethylene terephthalate glycol (PETG) vials of 60 mL were acquired from Thermo Fisher Scientific^TM^ Nalgene^TM^ (Waltham, MA, USA).

### 4.2. Fish Samples

**Figure 6:**
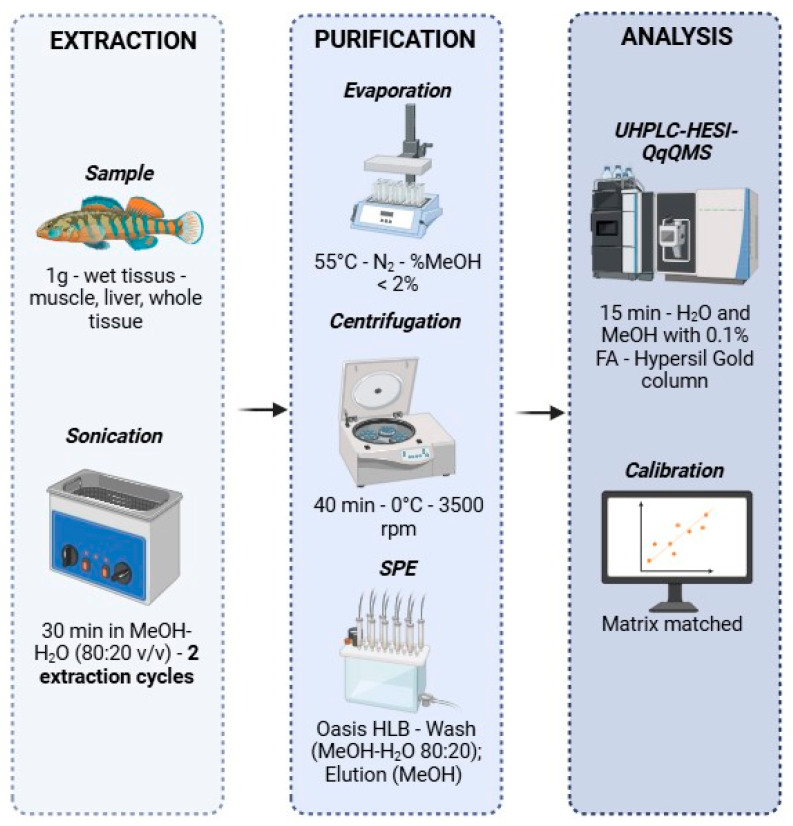
Optimal workflow for the analysis of 27 cyanopeptides in fish.

For method development, frozen sole muscle tissue samples were purchased from retail markets, while yellow perch and pike liver samples were obtained internally through collaboration with the Aquatic Contaminants Research Division of Environment and Climate Change Canada. Samples were selected based on availability and the absence of detectable cyanotoxins, serving as a baseline for comparison. Preliminary analyses confirmed that these samples were free of contamination by the target analytes. Four rainbow smelts (*Osmerus mordax*) were sampled on 12 November 2020, from Lac à la Perchaude (Shawinigan, QC, Canada) following a mass mortality event affecting this species. The sampling aimed to investigate potential causes, as no other fish species appeared impacted. Given the lake’s history of cyanobacterial blooms over the past three years, these samples were collected fresh and sent to the laboratory for analysis and stored at −20 °C. Samples were thawed at room temperature for 2 h before being homogenized with a Biohomogenizer from Biospec Products (Bartlesville, OK, USA). The optimal workflow is presented in Figure 6.

### 4.3. Extraction and Clean-Up

Analytes were extracted from liver and muscle tissues by ultrasonic-assisted extraction with an ultrasonic bath from Branson (Danbury, CT, USA). Glassware or PETG containers were preferred throughout all extraction and analysis steps due to the high sorption affinity of microcystins LW, LY, LA, and WR on polypropylene.

The optimal extraction conditions were: 1 g of thawed, ground wet matrix is placed in a 40 mL amber glass tube, and 10 mL of MeOH-H_2_O (80:20 *v*/*v*) is added. The mixture is vortexed for 30 s to enhance the contact between the extraction solvent and the matrix, then sonicated for 10 min and allowed to sediment for 10 min. The supernatant is collected and transferred to a 10 mL centrifuge tube. This process is then centrifuged with a Sorvall Legend RT+ centrifuge from Thermo Fisher Scientific (Pittsburgh, PA, USA) for 40 min at 3500 rpm and 0 °C. The supernatant is recovered, and these steps are repeated a total of three times for muscle tissue and two times for liver tissue.

After extraction, the samples were concentrated to approximately 5 mL under a nitrogen flow at 55 °C using RapidVap Vertex Dry Evaporators from Labconco (Kansas City, MO, USA). This step minimizes losses associated with the presence of MeOH. The samples were then centrifuged a second time for 30 min at −9 °C. This step facilitates the precipitation of proteins and lipids present in the sample, thereby enhancing the SPE loading and retention steps. The supernatant was subsequently diluted in 50 mL of deionized water in a 60 mL PETG bottle. This volume allows for the loading of SPE cartridges in approximately one hour, reduces the percentage of residual MeOH in the sample if present, and enables the use of sufficiently large volumes to limit losses due to dead volumes.

The samples were then purified by SPE following the method published by Roy-Lachapelle et al. [44]. Briefly, the cartridges were conditioned with 5 mL of MeOH and 5 mL of deionized water. The cartridges were loaded by gravity. Washing was performed with 5 mL of the MeOH-water mixture (80:20, *v*/*v*). The cartridges were eluted with 5 mL of MeOH. The samples were then evaporated to dryness under a nitrogen flow at 55 °C for 130 min and reconstituted in 1 mL of MeOH-water (8:92, *v*/*v*). The samples were vortexed for 30 s and sonicated for 1 min before being filtered through 0.22 µm filters, which have low sorption capacity (<10%) for analytes (Figure S4), prior to analysis by ultra-high liquid chromatography-triple quadrupole mass spectrometry (UHPLC-QqQMS).

### 4.4. Ultra-High Liquid Chromatography-Triple Quadrupole Mass Spectrometry (UHPLC-QqQMS)

The separation was performed on a Vanquish Horizon Binary UHPLC system equipped with a Hypersil Gold^TM^ column (150 × 2.1 mm, 1.9 µm particle size) from Thermo Fisher Scientific (Waltham, MA, USA) using an autosampler (set to 4 °C), a degasser, and a column heater (set at 40 °C). The sample injection volume and mobile phase flow rate were set to 10 µL and 400 µL min^−1^, respectively. The elution solvents used were water with 0.1% FA (solvent A) and acetonitrile with 0.1% FA (solvent B), with a 15-min gradient, as shown in Figure S7A. This system was coupled to a TSQ Altis™ Plus triple quadrupole mass spectrometer (Thermo Fisher Scientific, Waltham, MA, USA) equipped with a positive mode heated electrospray ionization (HESI) source, operated using Chromeleon 7.3 software (Thermo Fisher Scientific, Waltham, MA, USA). The HESI parameters included a spray voltage of 3900 V, sheath gas of 50 (arbitrary), auxiliary gas of 10 (arbitrary), sweep gas of 1 (arbitrary), ion transfer tube temperature of 325 °C and vaporizer temperature of 350 °C.

Data acquisition was conducted in selected reaction monitoring (SRM) mode. All MS parameters, including precursor ions, two product ions, collision energy, and retention time parameters, are detailed in Table S2 and were selected according to a previous study [44]. Data collection was managed using TraceFinder 5.1 software (Thermo Fisher Scientific, Waltham, MA, USA), and data processing was conducted in R (version 4.1.1) with the ggplot2 package (version 3.4.4).

### 4.5. Method Validation

The method’s performance was evaluated based on six analytical criteria: linearity of calibration curves, recovery, precision, and limits of detection and quantification. Quantification was carried out using a calibration curve in solvent with six calibration points (1, 5, 10, 20, 40, and 80 µg L^−1^) based on ordinary least squares linear regression. To account for signal and recovery variations, a homogenized matrix sample was spiked with 20 ng g^−1^ of the cyanotoxin mixture and equilibrated for 23 h to ensure proper binding (see Figure 4), after which it was processed following the developed extraction procedure. The calibration curve was then adjusted using a correction factor calculated according to the following Equation (1):

(1) $$Correction\;Factor\;\left(CF\right)=\frac{\left(A_{sample;20\;ppb}-A_{sample}\right)}{A_{solvent;20\;ppb}}$$

where A_sample_; 20 ppb and A_solvent_; 20 ppb are the chromatographic peak areas in the matrix and solvent spiked with a 20 ng g^−1^ cyanotoxin mixture, respectively. A_sample_ represents the chromatographic peak area of an unspiked sample extract.

The linear regression drift was assessed using two quantification controls at 2.5 µg L^−1^ (QC_low_) and 25 µg L^−1^ (QC_high_), injected immediately after the calibration curve and subsequently every 12 injections. The quality of the linear regression was evaluated based on the coefficient of determination (R^2^) and the residuals.

To determine the method recoveries, two sets of samples, referred to as Set A and Set B, were prepared identically except for the timing of the analyte spiking. For Set A, the analyte mixture in MeOH–H_2_O was spiked into 1 g of muscle or liver tissue (n = 3) to obtain the concentrations corresponding to QC_low_ and QC_high_. The samples were then vigorously mixed for 1 min to promote contact between the matrix and the analytes. Set A was stored in the dark at 4 °C for at least 12 h prior to extraction to allow equilibration between the analytes and the matrix.

For Set B (n = 3), the analytes were spiked after the evaporation step. Recoveries were calculated by comparing the signal areas from Sets A and B. This approach compensates for matrix effects and potential matrix contamination that could affect quantification accuracy.

The precision and accuracy of the method were evaluated using the same Set A employed for determining method recoveries. Method precision was assessed based on the standard deviation and the mean of the concentrations derived from Set A. Accuracy was determined as the percentage bias between the mean concentrations obtained from Set A and theoretical concentrations.

The limits of detection (LOD) and quantification (LOQ) of the method were calculated using the linear regression approach. These coefficients of merit were determined by dividing the standard deviation of the signal at the lowest concentration (n = 10) by the slope of the calibration curve. This value was then multiplied by a factor of 3 for the LOD and by a factor of 10 for the LOQ.

Matrix effects were evaluated by comparing the peak areas of Set B at QC_low_ and QC_high_ to the peak areas of QC_low_ and QC_high_ in the solvent, using the following Equation (2):

(2) $$Matrix\;effect\;\left(\%\right)=\left[\left(\frac{A_{sample}}{A_{solvant}}\right)\times 100\right]-100$$

where A_matrix_ represents the peak area of the analyte in the matrix (Set B), and A_solvent_ represents the peak area of the analyte in the solvent.

## Data Availability

All original contributions of this study are contained within the article. For additional information, please contact the corresponding author.

## Supplementary Materials

The following supporting information can be downloaded at: https://www.mdpi.com/article/10.3390/toxins17120580/s1, Figure S1: Representative structures of the target cyanopeptide families; Figure S2: Cyanopeptide losses induced by using polypropylene vials in distilled water spiked at 10 µL^−1^; Figure S3: Cyanopeptide recoveries induced by using polypropylene vials in muscle matrix spiked at 10 ng g^−1^; Figure S4: Recovery of the 0.22 µm filtration step. Deionized water samples were spiked at a concentration of 10 µL^−1^ and filtered once; Figure S5: *p*-value (at 95%) of SPE losses induced by the addition of 5% MeOH. A deionized water sample (100 mL) was spiked at a concentration of 10 ppb. The colors represent the significance of data, green for *p*-value > 0.05 and red for *p*-value < 0.05; Figure S6: Recovery of the 0.22 µm filtration step. Deionized water samples were spiked at a concentration of 10 µL^−1^ and filtered once; Figure S7: Optimization of the chromatographic gradient. In Figure A, the color scheme distinguishes the newly optimized gradient conditions (blue) from the original ones (orange). Figure B highlights the impact of gradient conditions on chromatographic peak shape; Figure S8: Evaluation of the impact of different chromatographic gradient conditions on matrix effects; Figure S9: Calibration curves established in solvent, in matrix, and in matrix after application of the correction factor (CF); Table S1: Coefficient of determination (R^2^) values for calibration curves established in solvent, in matrix, and in matrix after application of the correction factor (CF); Table S2: MS parameters for targeted compounds quantitative analysis.

## Abbreviations

The following abbreviations are used in this manuscript:

Adda	3-Amino-9-methoxy-2,6,8-trimethyl-10-phenyl-deca-4,6-dienoic acid
MMPB	2-Methyl-3-methoxy-4-phenylbutyric acid
MC	Microcystin
PP	Polypropylene
SPE	Solid-phase extraction
ANOVA	Analysis of variance
MG	Microginin
AGU	Aeruginoguanidine
AP	Anabaenopeptin
FA-A	Ferintoic acid A
MeOH	Methanol
logD	Distribution coefficient
ACN	Acetonitrile
GHP	Hydrophilic polypropylene
Mdha	*N*-methyldehydroalanine)
TR	Retention time
QC	Quality control
RSD	Relative standard deviations
R^2^	Coefficient of determination
LOQ	Limit of quantification
MQL	Method limit of quantification
LOD	Limit of detection
MDL	Method limit of detection
FA	Formic acid
PETG	Polyethylene terephthalate glycol
UHPLC-QqQMS	Ultra-high liquid chromatography-triple quadrupole mass spectrometry
HESI	Heated electrospray ionization
SRM	Selected reaction monitoring
CF	Correction factor
