# Supplementary material for: Overcoming Analytical Challenges for the Detection of 27 Cyanopeptides Using a UHPLC-QqQ-MS Method in Fish Tissues
Source: Toxins (Basel). 2025 Dec 2;17(12):580. doi: 10.3390/toxins17120580 (PMC12737578; doi:10.3390/toxins17120580)
Supplement: Supplementary file 1 [file toxins-17-00580-s001.zip › toxins-3983389-supplementary.pdf]

SUPPLEMENTARY MATERIALS

Page S-2, Figure S1. Representative structures of the target cyanopeptide families.

Page S-3, Figure S2. Cyanopeptides losses induced by using polypropylene vials in distilled water spiked at 10 µ L^{-1}.

Page S-3, Figure S3. Cyanopeptides recoveries induced by using polypropylene vials in muscle matrix spiked at 10 ng g^{-1}.

Page S-4, Figure S4. Recovery of the 0.22 µm filtration step. Deionized water samples were spiked at a concentration of 10 µ L^{-1} and filtered once.

Page S-4, Figure S5. P-value (at 95%) of SPE losses induced by the addition of 5% MeOH. Deionized water sample (100 mL) spiked at a concentration of 10 ppb. The colours represent the significance of data, green for p-value > 0.05 and red for p-value < 0.05.

Page S-5, Figure S6. Recovery of the 0.22 µm filtration step. Deionized water samples were spiked at a concentration of 10 µ L^{-1} and filtered once.

Page S-5, Figure S7. Optimization of the chromatographic gradient. In Figure A, the color scheme distinguishes the newly optimized gradient conditions (blue) from the original ones (orange). Figure B highlights the impact of gradient conditions on chromatographic peak shape.

Page S-6, Figure S8. Evaluation of the impact of different chromatographic gradient conditions on matrix effects.

Page S-6, Figure S9. Calibration curves established in solvent, in matrix, and in matrix after application of the correction factor (CF).

Page S-7, Table S1. Coefficient of determination (R^2) values for calibration curves established in solvent, in matrix, and in matrix after application of the correction factor (CF).

Page S-8, Table S2. MS parameters for targeted compounds quantitative analysis.

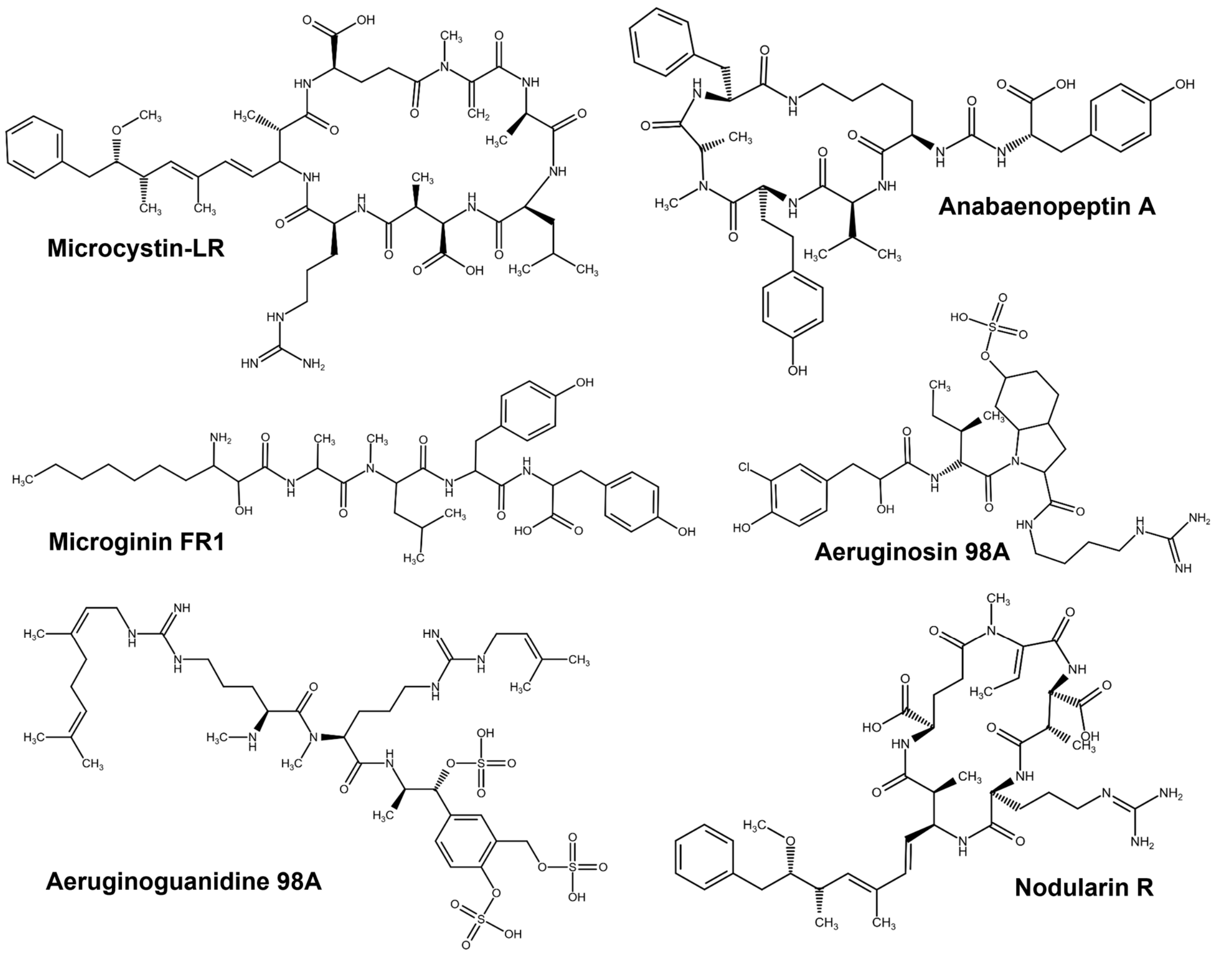

Figure S1. Representative structures of the target cyanopeptide families.

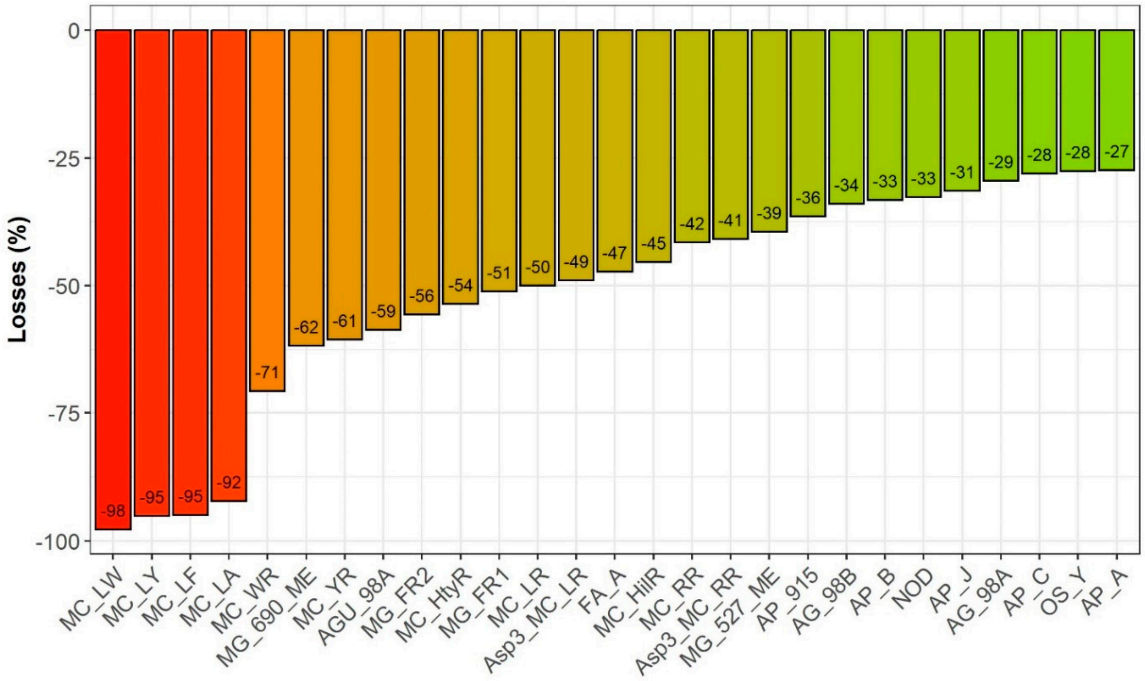

Figure S2. Cyanopeptides losses induced by using polypropylene vials in distilled water spiked at 10 µ L⁻¹.

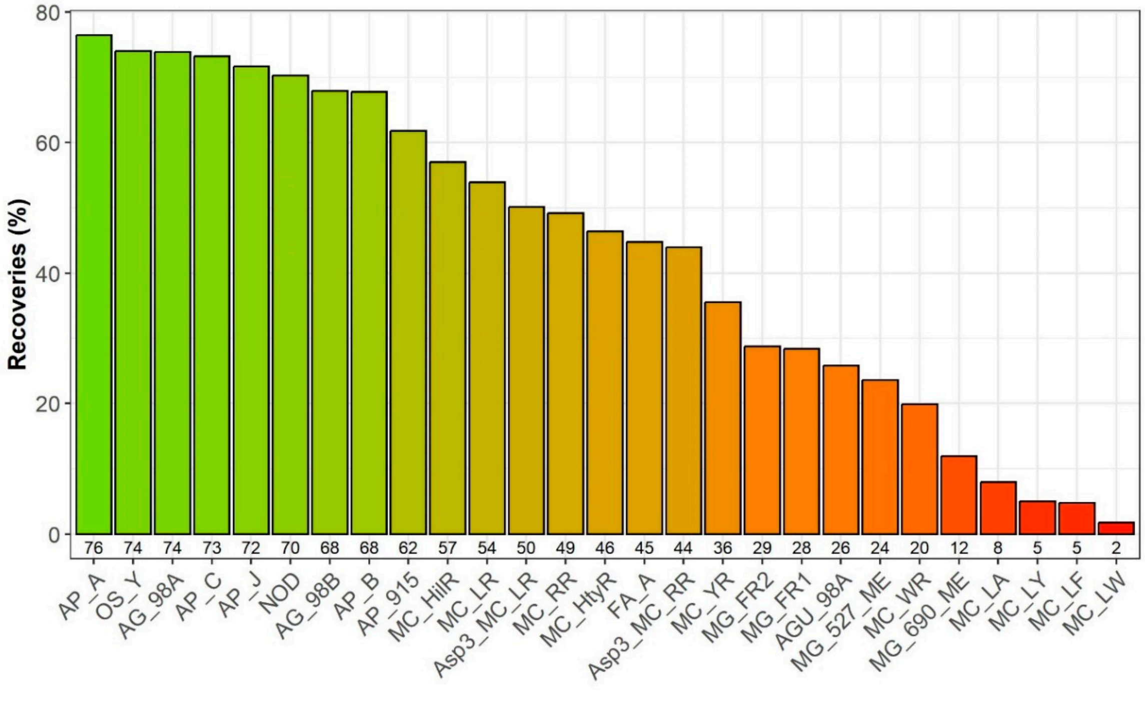

Figure S3. Cyanopeptides recoveries induced by using polypropylene vials in muscle matrix spiked at 10 ng g^{-1}.

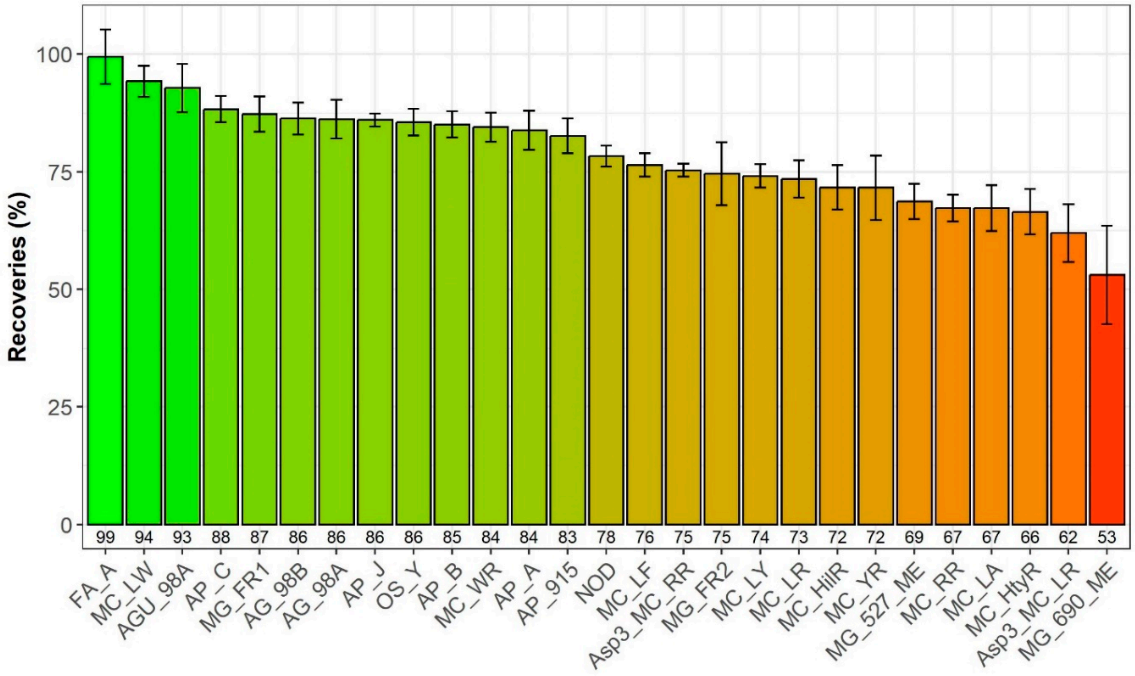

Figure S4. Evaluation of cyanotoxin recoveries resulting from liquid–liquid extraction in hexane, performed on muscle tissue matrix spiked at 10 ng g^{-1}.

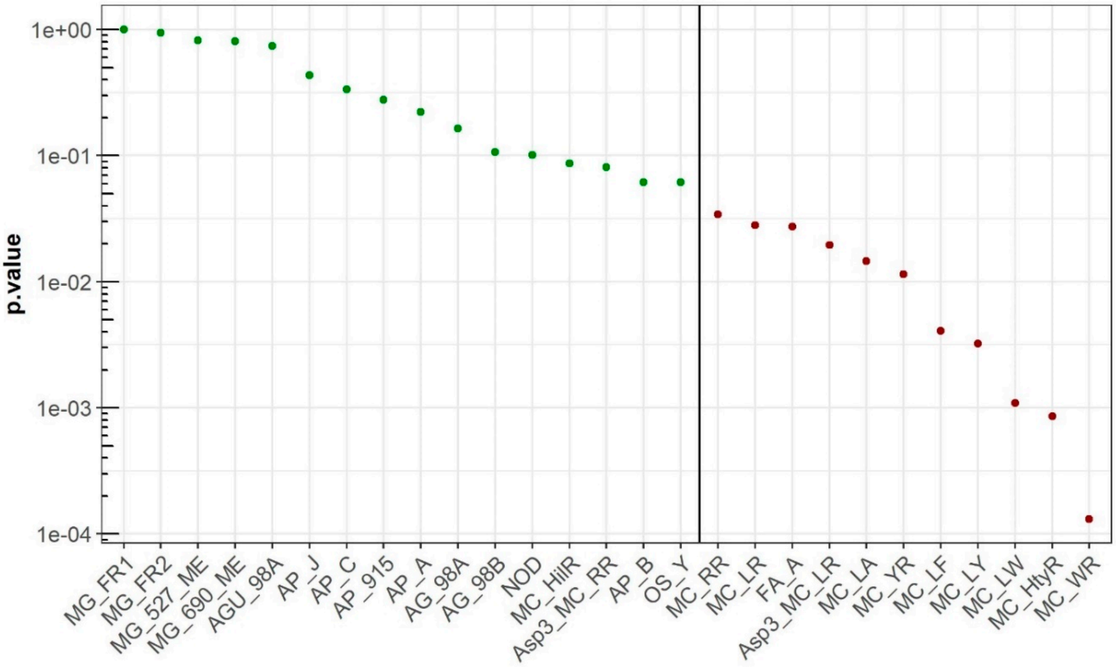

Figure S5. P-value (at 95%) of SPE losses induced by the addition of 5% MeOH. Deionized water sample (100 mL) spiked at a concentration of 10 ppb. The colours represent the significance of data, green for p-value > 0.05 and red for p-value < 0.05.

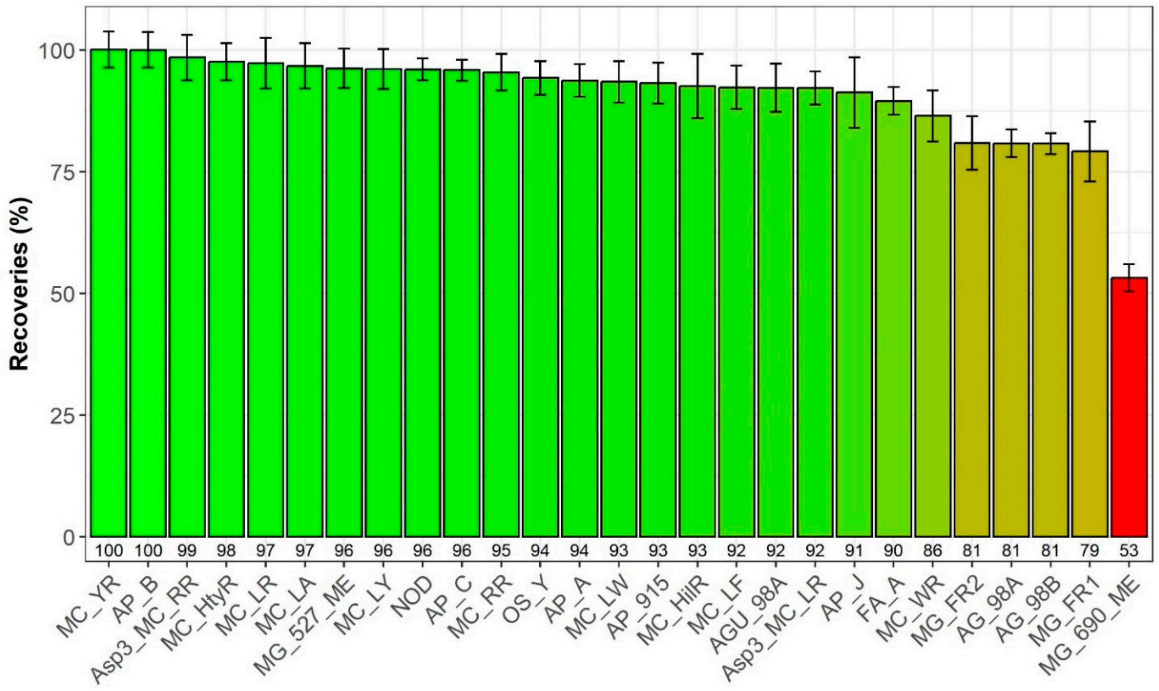

Figure S6. Recovery of the 0.22 µm filtration step. Deionized water samples were spiked at a concentration of 10 µ L^{-1} and filtered once.

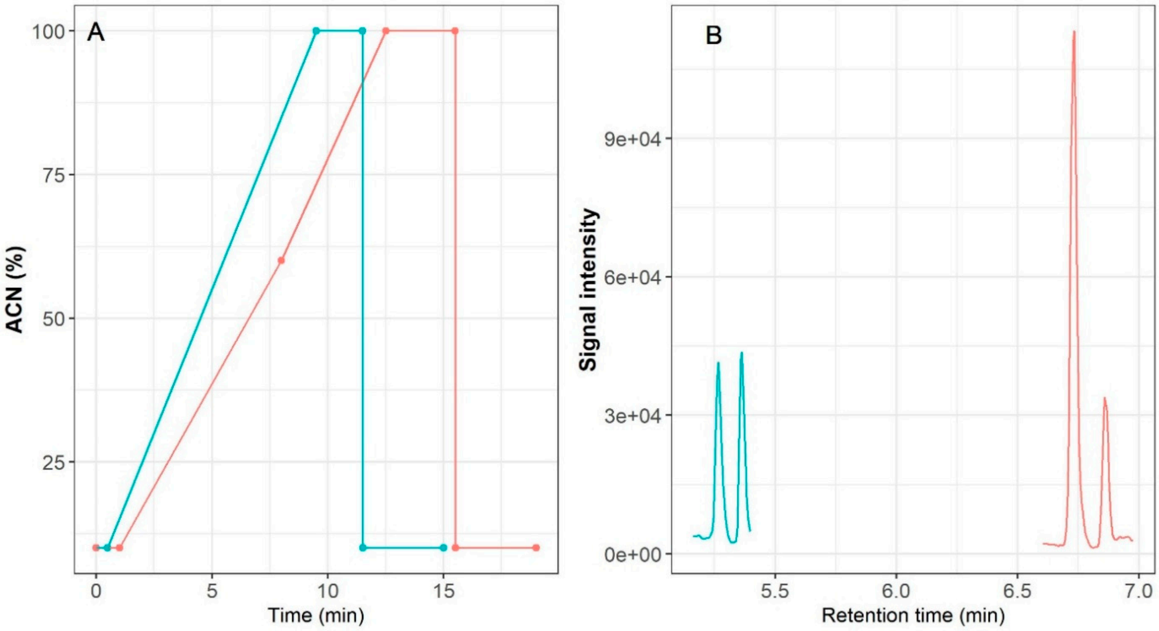

Figure S7. Optimization of the chromatographic gradient. In Figure A, the color scheme distinguishes the newly optimized gradient conditions (blue) from the original ones (orange). Figure B highlights the impact of gradient conditions on chromatographic peak shape.

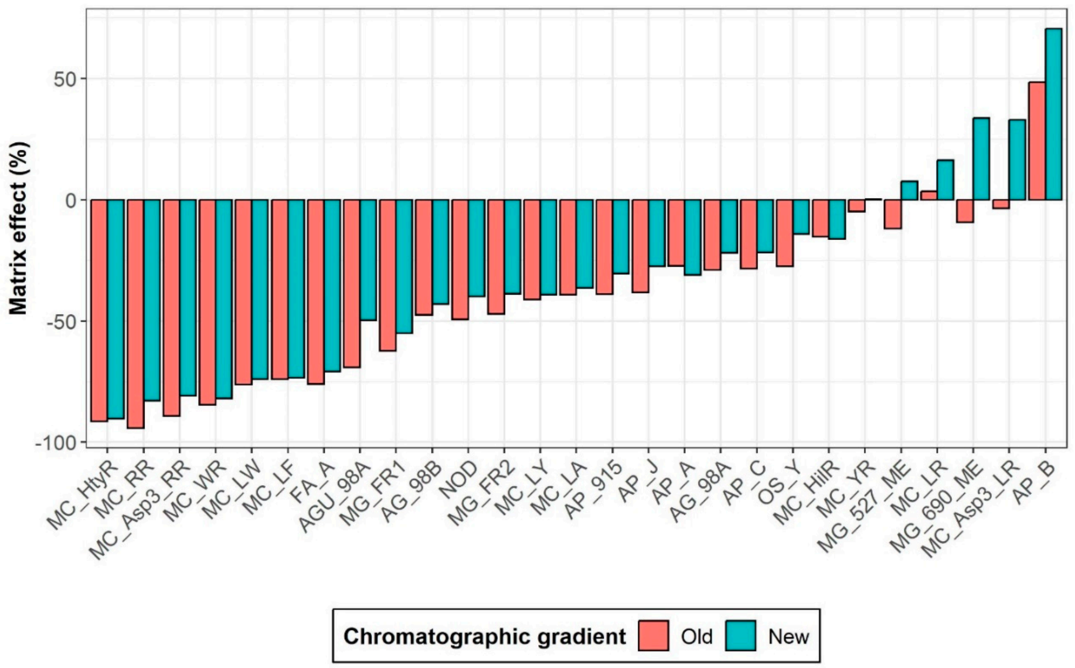

Figure S8. Evaluation of the impact of different chromatographic gradient conditions on matrix effects.

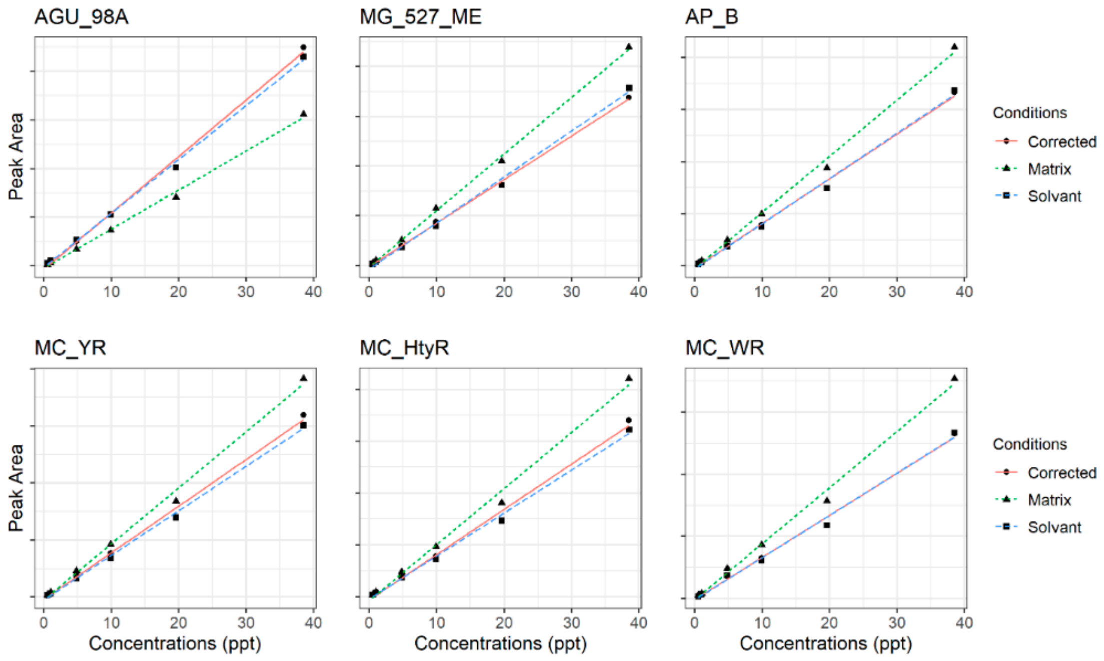

Figure S9. Calibration curves established in solvent, in matrix, and in matrix after application of the correction factor (CF).

Table S1. Coefficient of determination (R^2) values for calibration curves established in solvent, in matrix, and in matrix after application of the correction factor (CF).

Class	Compounds	Matrix	Solvent	Matrix corrected
Aeruginosins	AG-98A	0.997	0.998	0.997
	AG-98B	0.996	0.998	0.996
Aeruginoguanidine	AGU-98A	0.997	0.998	0.997
Anabaenopeptins	AP-915	0.994	0.997	0.994
	AP-A	0.996	0.996	0.996
	AP-B	0.996	0.995	0.996
	AP-C	0.996	0.996	0.996
	AP-J	0.995	0.996	0.995
	FA-A	0.995	0.995	0.995
	OS-Y	0.994	0.997	0.994
Microcystins	Asp3-MC-LR	0.995	0.996	0.995
	Asp3-MC-RR	0.993	0.999	0.993
	MC-HilR	0.996	0.997	0.996
	MC-HtyR	0.994	0.996	0.994
	MC-LA	0.995	0.996	0.995
	MC-LF	0.996	0.995	0.996
	MC-LR	0.994	0.997	0.994
	MC-LW	0.997	0.996	0.997
	MC-LY	0.995	0.997	0.995
	MC-RR	0.996	0.997	0.996
	MC-WR	0.995	0.994	0.995
	MC-YR	0.995	0.998	0.995
Microginins	MG-527-ME	0.999	0.997	0.999
	MG-690-ME	0.997	0.995	0.997
	MG-FR1	0.996	0.996	0.996
	MG-FR2	0.997	0.995	0.997
Nodularin	NOD	0.997	0.995	0.997

Table S2. MS parameters for targeted compounds quantitative analysis

Compounds	Retention Time (min)	Retention Time Window (min)	Precursor ion (*m/z*)	Product ion (*m/z*)*	Collision Energy (V)	RF Lens (V)
AG-98A	4.40	0.45	655.3	281.1	40	87
				575.2	18	
AG-98B	5.00	0.45	689.2	281.2	41	89
				609.2	18	
AP-B	6.00	0.45	837.4	175.1	38	127
				201.1	42	
MG-527-ME	6.30	0.45	542.3	102	35	97
				194	23	
[Asp^3]MC-RR	6.67	0.45	512.9	445.7	20	92
				620.2	27	
MG-690-ME	6.78	0.61	705.3	357.1	25	103
				510.2	20	
AGU-98A	6.53	0.45	982.4	804.4	27	94
				902.4	10	
MC-RR	6.80	0.38	519.9	135.1	31	99
				213.1	36	
MC-RR-$^{15}N_{13}$	6.80	0.38	526.1	135.1	31	99
AP-A	6.95	0.45	844.4	637.2	26	121
				663.2	25	
AP-915	7.10	0.45	916.4	709.4	29	131
				735.3	28	
NOD-R	7.22	0.47	825.5	135.1	59	128
				389.2	42	
OC-Y	7.23	0.45	858.4	651.3	23	145
				1024.5	15	
FA-A	7.28	1.25	867.4	637.3	28	123
				686.2	37	
MG-FR1	7.30	0.48	728.4	384.2	23	112
				664.2	32	
MC-YR	7.43	0.45	1045.5	135	71	120

				1017.2	45	
MC-YR-$^{15}N_{10}$	7.43	0.45	1055.6	215.1	65	120
MC-HtyR	7.48	0.45	1059.5	107.1	85	120
				135.1	70	
MG-FR2	7.48	0.44	742.4	182.1	43	123
				398.2	25	
[Asp^3]MC-LR	7.57	0.47	981.5	135.1	66	126
				539.2	48	
MC-LR	7.57	0.47	995.5	135.1	70	122
				213.1	64	
MC-LR-$^{15}N_{10}$	7.57	0.46	1005.1	215.1	65	122
MC-HilR	7.75	0.45	1009.5	213.1	77	101
				269.1	57	
MC-WR	7.82	0.45	1068.5	135.1	70	116
				213.1	64	
AP-C	7.90	0.45	808.4	419.2	37	117
				651.3	25	
AP-J	8.28	1.45	794.4	637.2	24	107
				663.3	24	
MC-LA	8.93	0.5	910.4	375.2	32	113
				776.3	19	
MC-LA-$^{15}N_7$	8.93	0.5	917.5	215.1	65	131
MC-LY	9.06	0.45	1002.5	494.2	26	118
				868.4	20	
MC-LW	10.00	1	1025.5	517.2	27	140
				891.4	21	
MC-LF	10.14	0.72	986.5	478.2	25	144
				852.4	20	

*The first product ion is for quantification, and the second product ion is for confirmation
